# Identification of immune-related and autophagy-related genes for the prediction of survival in bladder cancer

**DOI:** 10.1186/s12863-022-01073-7

**Published:** 2022-08-01

**Authors:** Quanfeng Zhu, Lingdi Zhang, Yaping Deng, Leilei Tang

**Affiliations:** Department of Pharmacy, Affiliated Xiaoshan Hospital, Hangzhou Normal University, Hangzhou, 311201 China

**Keywords:** Bladder cancer, Immune, Autophagy, Risk model, Prognosis

## Abstract

**Background:**

Bladder cancer has the characteristics of high morbidity and mortality, and the prevalence of bladder cancer has been increasing in recent years. Immune and autophagy related genes play important roles in cancer, but there are few studies on their effects on the prognosis of bladder cancer patients.

**Methods:**

Using gene expression data from the TCGA-BLCA database, we clustered bladder cancer samples into 6 immune-related and autophagy-related molecular subtypes with different prognostic outcomes based on 2208 immune-related and autophagy-related genes. Six subtypes were divided into two groups which had significantly different prognosis. Differential expression analysis was used to explore genes closely related to the progression of bladder cancer. Then we used Cox stepwise regression to define a combination of gene expression levels and immune infiltration indexes to construct the risk model. Finally, we built a Nomogram which consist of risk score and several other prognosis-related clinical indicators.

**Results:**

The risk model suggested that high expression of C5AR2, CSF3R, FBXW10, FCAR, GHR, OLR1, PGLYRP3, RASGRP4, S100A12 was associated with poor prognosis, while high expression level of CD96, IL10, MEFV pointed to a better prognosis. Validation by internal and external dataset suggested that our risk model had a high ability to discriminate between the outcomes of patients with bladder cancer. The immunohistochemical results basically confirmed our results. The C-Index value and Calibration curves verified the robustness of Nomogram.

**Conclusions:**

Our study constructed a model that included a risk score for patients with bladder cancer, which provided a lot of helps to predict the prognosis of patients with bladder cancer.

**Supplementary Information:**

The online version contains supplementary material available at 10.1186/s12863-022-01073-7.

## Introduction

Bladder cancer (BLCA) is a high-incidence tumor which has high morbidity and mortality. According to statistics, BLCA ranks ninth in the prevalence of malignant diseases and 13th among the most common causes of cancer death [1].

For the past decades, BLCA has made many advances in clinical treatment. Gene sequencing technology has identified the most mutated genes in BLCA. Many studies have researched the treatment of BLCA at the level of cells and molecular mechanism [2]. Meanwhile, the recent emergence of immunotherapy brings new dawn to the treatment of bladder cancer [3].

Most of the immunomodulatory effects of immunotherapy are achieved by enhancing T cell responses. Although immunotherapy has brought many new breakthroughs to the treatment of tumors, only a small proportion of patients profit from it, which highlight the necessity of identifying new cells and new molecules [4, 5]. For example, some researchers explored the immunotherapy of hepatocellular carcinoma by typing immune cells [6].

In many cancers, the regulations of autophagy play important roles. Autophagy plays dynamic inhibitory or promotion effects in different stages of tumors. Therefore, understanding how autophagy regulates metabolism and tumor growth is essential for tumor treatment [7, 8].

In recent years, methods of bioinformatics and data mining have been increasingly applied to various medical researches. In the field of oncology, researchers have achieved many clinically meaningful results using these methods. Xie et al. found and confirmed the significant role of ITPA in uveal melanoma through bioinformatics analysis and cell experiments, which provided a certain reference for the diagnosis and treatment of uveal melanoma [9].

In the present study, immune genes and autophagy genes were collected. We utilized gene expression data from two commonly used databases (The Cancer Genome Atlas (TCGA) and Gene Expression Omnibus (GEO)) to construct BLCA molecular subtypes based on immune-related and autophagy-related genes. It showed that these subtypes distinguished well among patients with different prognosis of BLCA [10, 11]. Through differential gene expression analysis, immune infiltration analysis and Cox stepwise regression, we finally construct a prognostic risk model with 12 genes and 3 immune infiltrating cell types which were screened out from 65 differentially expressed genes. Our model was validated by both internal and external validation cohorts and could provide an important reference for clinicians to predict the prognosis of patients with BLCA.

## Materials and methods

### Data preparation

The TCGA-BLCA dataset included the RNA-seq data of 410 BLCA and 37 adjacent normal samples was downloaded from the TCGA database (https://portal.gdc.cancer.gov/). The gene expression data consisted of 166, 116 BLCA samples from GSE13507 and GSE48276 datasets were downloaded from GEO database (http://ncbi.nlm.nih.gov/geo/), respectively. We conducted preliminary screening of the downloaded original data according to the following criteria: (1) genes with zero expression in more than 30% of the samples were excluded; (2) excluding the genes whose expression values were lost; (3) excluding the samples without related clinical data; (4) excluding the samples with OS < 30 days; (5) excluding non-tumor tissue samples. We divided the TCGA-BLCA dataset into two random cohorts according to the ratio of 3 to 1: training cohort (*n* = 307) and internal validation dataset (*n* = 102). GSE13507 and GSE48276 datasets were regarded as external validation cohorts. Then, we downloaded 2208 human immune genes and autophagy genes from the Reactome database (https://reactome.org/) [12].

### Molecular subtypes

Based on the 2208 genes selected above, those screened training cohort samples were clustered according to the non-negative matrix factorization (NMF) in R package “NMF”. Moreover, we used Cibersort to calculate the immune infiltration scores of the subtypes for molecular subtype analysis [13].

### DEG identification and function and pathway enrichment analysis

We utilized the R package “DESeq2” to compute the differentially expressed genes (DEGs) according to the criteria FDR < 0.05 and FC > 1.5. Gene Ontology (GO) Term Enrichment Analysis and Kyoto Encyclopedia of Genes and Genomes (KEGG) Pathway Analysis were conducted based on the DEGs [14–16]. Moreover, Since GSEA analysis can better explain the enrichment of pathways, we conducted GSEA KEGG enrichment analysis on all genes after sequencing them according to FC from large to small (*P* < 0.05 and FDR < 0.25).

### Risk model construction and validation

We used above DEGs expression data to build a risk score model. Univariate Cox survival analysis in R studio was used to analyze the correlation between each DEG, immune infiltration score and OS of the BLCA patients. Wald test *P* < 0.05 was considered significant statistically. Then, we utilized Cox stepwise regression to further narrow the variables obtained above. Finally, a risk score model including variables weighted by their Cox stepwise coefficients was established. According to the formula: risk score = h0(t)(Value_variable1_*β_variable1_+ Value_variable1_*β_variable1_+ … … + Value_variable(n)_*β_variable(n)_), we figured up the risk score for each sample, separately. A TCGA-BLCA internal verification cohort and two GEO external verification cohorts were used to verify the credibility of the model, respectively. In order to further evaluate the robustness of the model, we used the R package of “pROC” to plot the risk score distribution of each cohort according to the risk score of each sample. According to the different risk scores of samples, samples with scores greater than zero were put in high-risk group, samples with scores less than zero were put in low-risk group. The Kaplan-Meier (KM) survival curve was used to perform the OS of each group and the log-rank test was used to compare the survival differences between the samples of high-risk group and low-risk group.

## Results

### Molecular subtypes

**Fig. 1:**
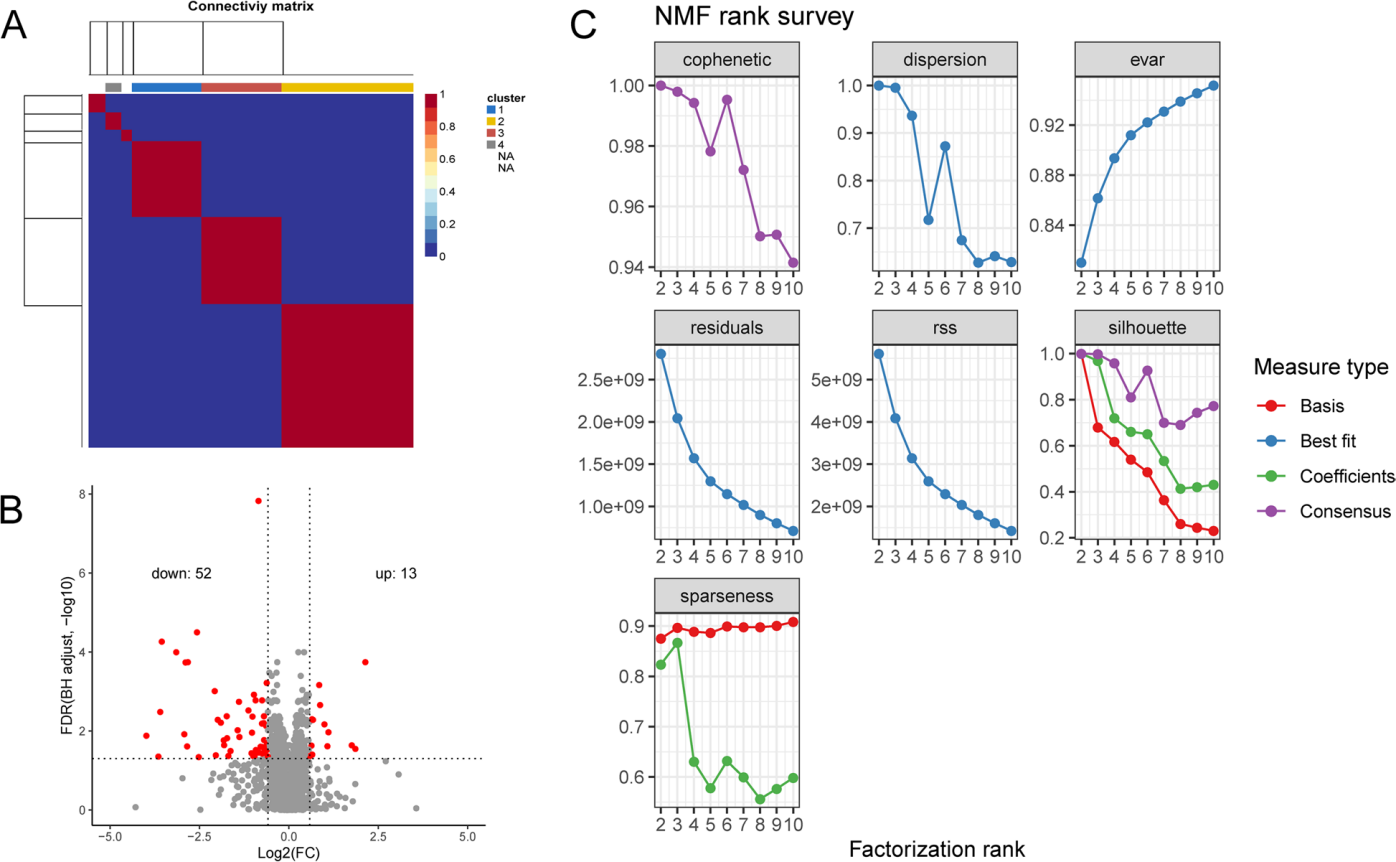
Identification of molecular subtypes of bladder cancer (BLCA) using TCGA data associated with prognosis. **A-B** The non-negative matrix factorization (NMF) consensus matrix plot for 2208 immune-related and autophagy-related genes identified six distinct BLCA subtypes. **C** Volcano plots of differentially expressed genes (DEGs) in two subtypes distinguished by prognosis

**Fig. 2:**
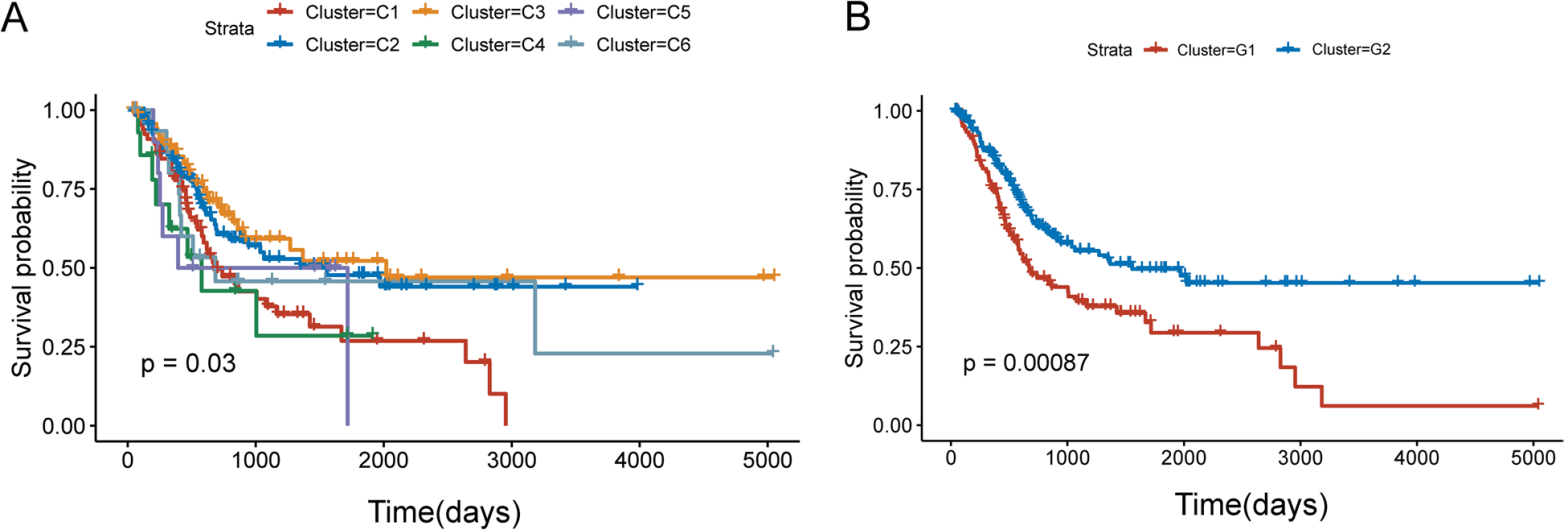
Prognostic analysis between different subtypes. **A** Kaplan–Meier (KM) curves of overall survival (OS) in the six molecular subtypes. **B** KM curves of OS in the two subtypes distinguished by prognosis

After NMF cluster of the training cohort samples, we screened out the best clustering digit: 6, based on cophenetic and dispersion (Fig. 1A and B). The prognosis of samples in 6 subtypes was further analyzed. The prognosis of C2 and C3 subtypes was better than other subtypes (Fig. 2A). For further analysis, we combined C2 and C3 subtypes into one subtype (G1), and other subtypes into another subtype (G2). As shown in Fig. 2B, there was a significant difference in prognosis between G1 and G2 (*P* < 0.001). We analyzed the pTNM staging of patients in high-risk group and low-risk group. After excluding patients without pTNM staging and TX, NX, and MX staging, we found no significant difference in pTNM staging between the two groups. (*P* > 0.05) (Table S1). To explore whether there were differences in immunity between high-risk and low-risk patients, we made comparative statistics on the immune infiltration indexes of high risk and low risk groups, and found that the indexes of T cells CD4 memory activated, Macrophages M0, Dendritic cells activated, Mast cells resting and Eosinophils were significantly different (*P* < 0.01) (Table S2). T cells CD4 memory activated and Dendritic cells activated in high-risk group were significantly lower than those in low-risk group, while Macrophages M0, Mast cells resting and Eosinophils were significantly higher than those in low-risk group. This reflected the differences between the two groups of patients in the immune microenvironment, which could provide a certain reference for exploring the immunotherapy of BLCA. In terms of neoadjuvant treatment, neoadjuvant chemotherapy can improve the prognosis of BLCA patients by enhancing the anti-tumor immune response [17]. In our research, only 4 patients in high-risk group and 2 patients in low-risk group received neoadjuvant treatment. Therefore, in this study, whether or not to receive neoadjuvant treatment did not have a significant impact on the prognosis of patients.

### DEG identification and bioinformatics analysis

**Fig. 3:**
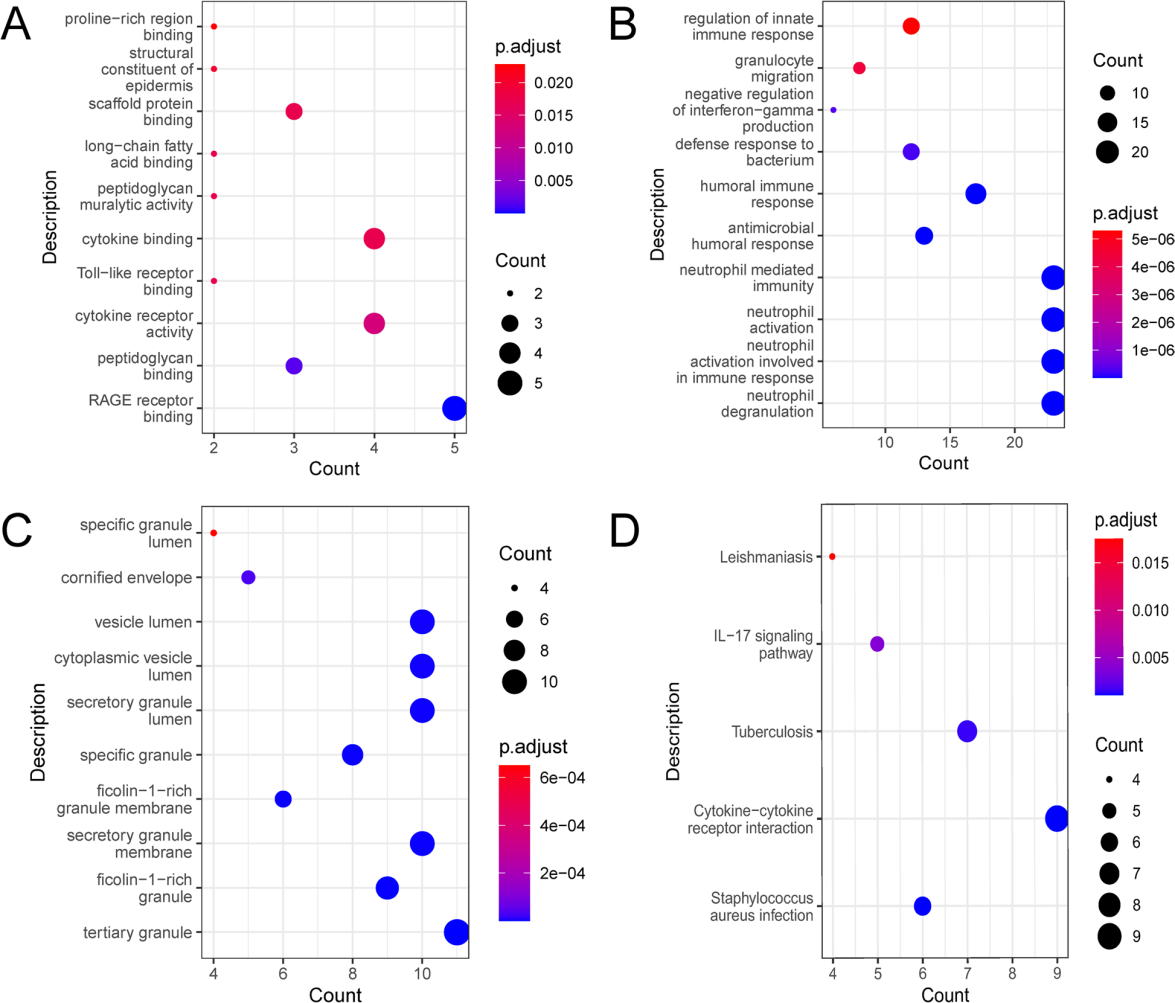
Functional enrichment analysis of differentially expressed genes (DEGs). **A-C** Enrichment molecular function (**A**), biological process (**B**), and cellular component (**C**) of DEGs. **D **Kyoto Encyclopedia of Genes and Genomes (KEGG) pathway enrichment analysis of DEGs

From 2208 immune-related and autophagy-related genes (Table S3), a total of 65 DEGs (Table S4, Fig. 1C) were identified between the two subtypes. Then, these DEGs were analyzed for GO and KEGG enrichment. In the enrichment results of molecular function (MF), biological process (BP) and cellular component (CC), the terms in the forefront are listed in Fig. 3A-Fig. 3C. MF analysis displayed the DEGs were particularly enriched in RAGE receptor binding and cytokine receptor binding (Fig. 3A). BP analysis demonstrated that the DEGs were basically enriched in neutrophil degranulation, neutrophil activation involved in immune response, neutrophil activation and neutrophil mediated immunity (Fig. 3B). CC analysis showed the DEGs were mainly enriched in tertiary granule, secretory granule membrane, secretory granule lumen, cytoplasmic vesicle lumen and vesicle lumen (Fig. 3C). KEGG analysis results showed that the main pathways involved in DEGs were Cytokine-cytokine receptor interaction, Tuberculosis and *Staphylococcus aureus* infection (Fig. 3D). GSEA KEGG enrichment analysis showed that genes were enriched in Epstein-Barr Virus Infection and Herpes Simplex Virus 1 Infection and were upward (Table S5).

### Establishment of prognostic risk model and immunohistochemical confirmation

**Fig. 4:**
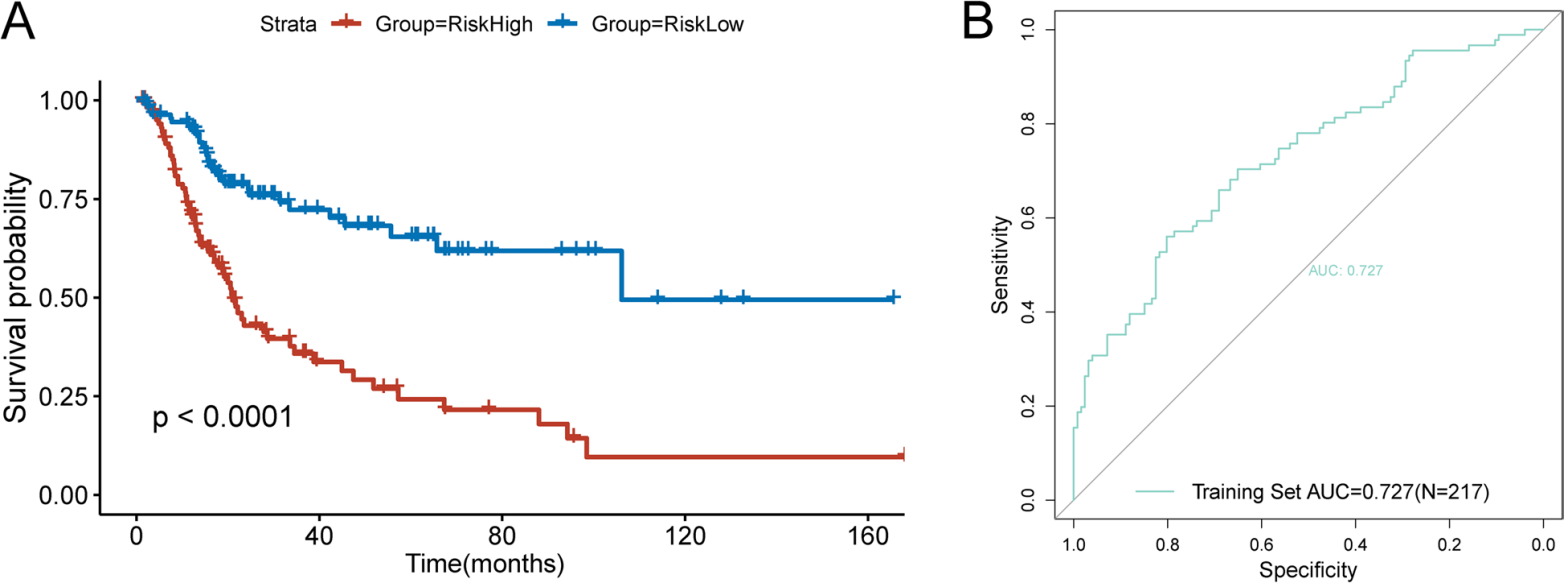
Evaluation of the performance of the risk model in the training cohort. **A** KM curves of the OS in the training cohort. **B** ROC curves and area under the curve (AUC) in the training cohort of the risk model

**Fig. 5:**
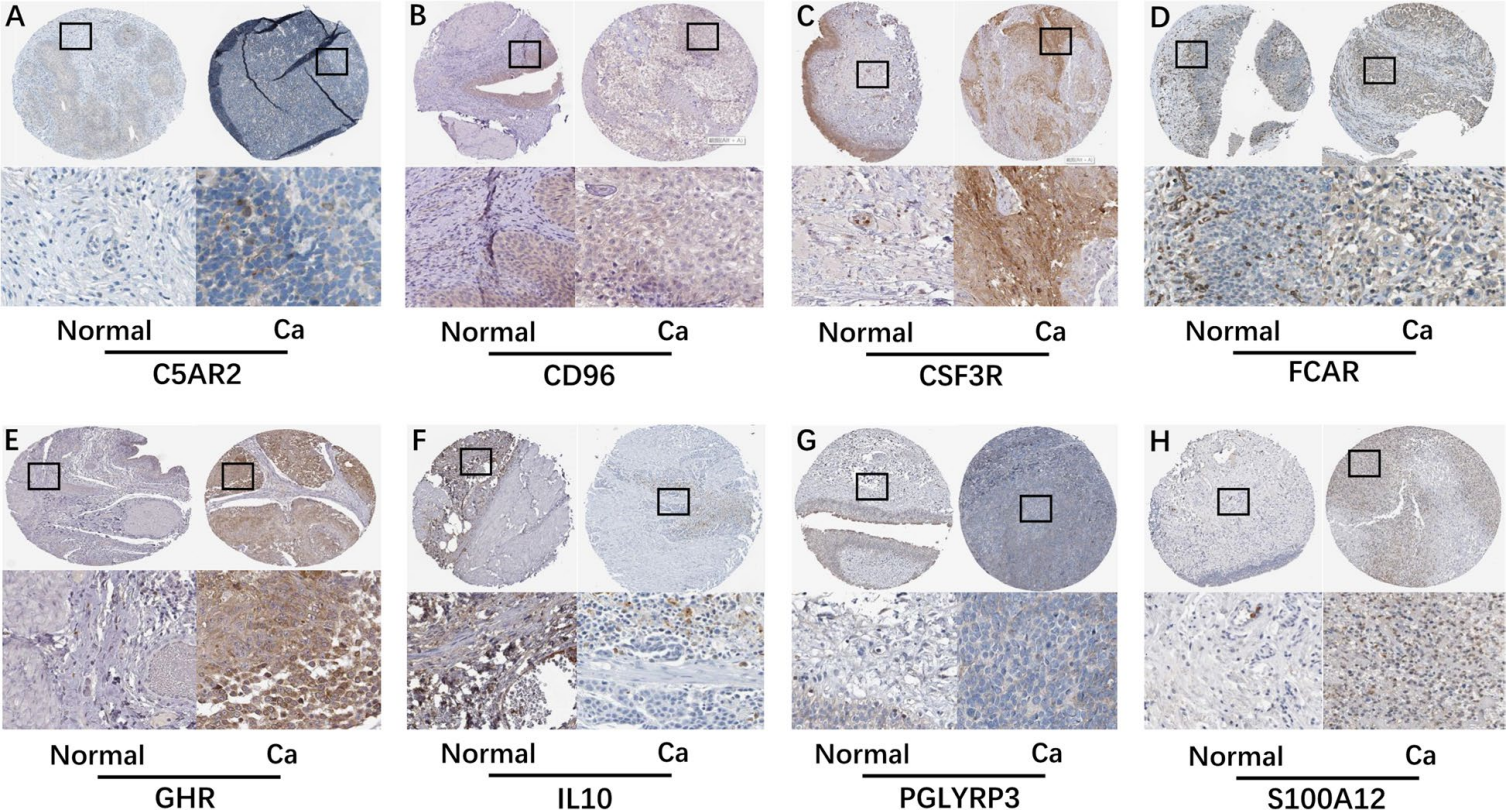
The results of immunohistochemistry in eight genes. **A, C, D, E, G, H** C5AR2, CSF3R, FCAR, GHR, PGLYRP3 and S100A12 were highly expressed in BLCA tissues. **B, F** CD96 and IL10 were lowly expressed in BLCA tissues

**Fig. 6:**
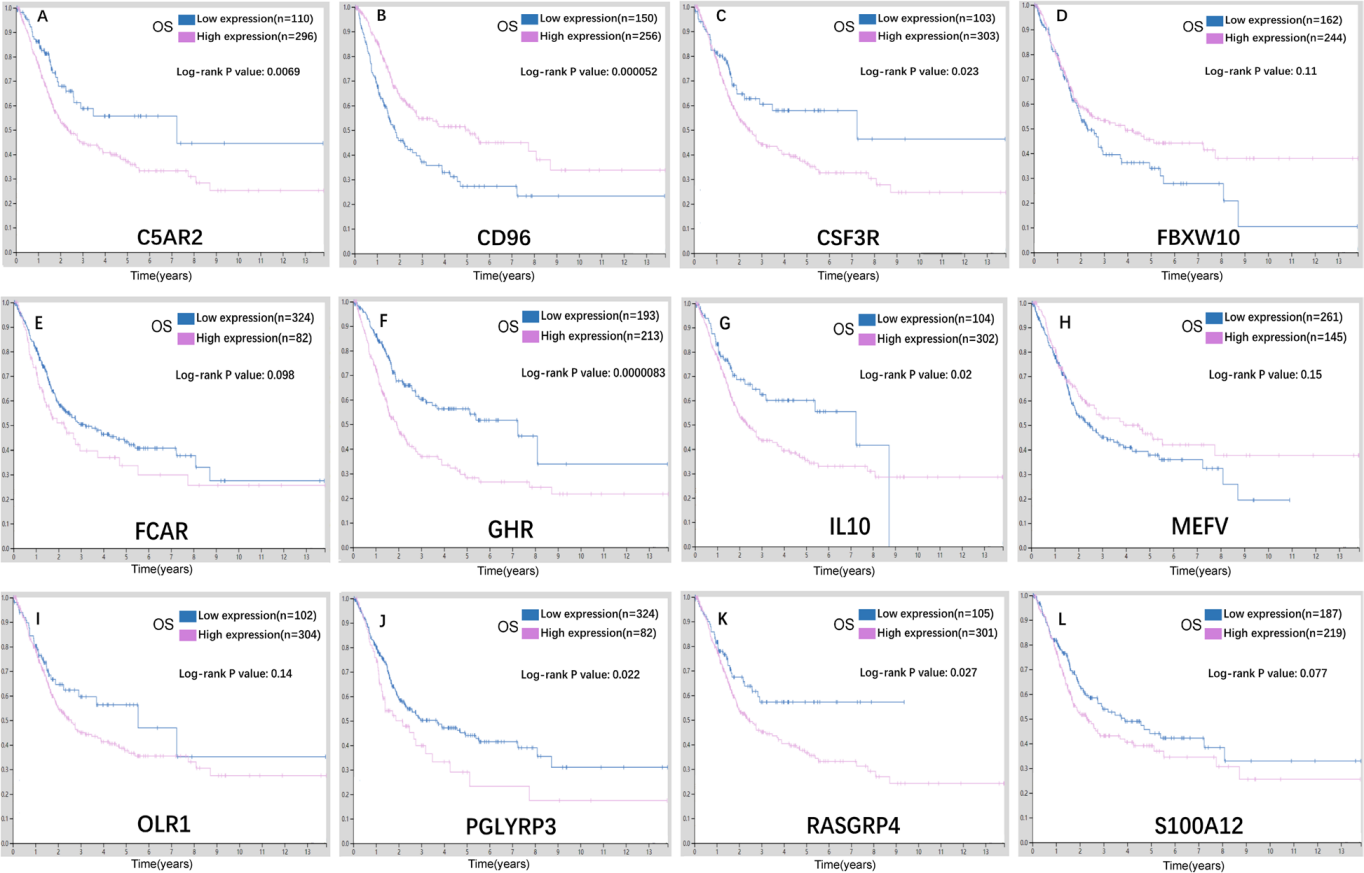
Relationships between gene expressions and prognosis of BLCA patients from Human Protein Atlas (HPA). **A-L** Relationships between expression levels of C5AR2, CD96, CSF3R, FBXW10, FCAR, GHR, IL10, MEFV, OLR1, PGLYRP3, RASGRP4, S100A12 and prognosis of BLCA patients

According to the training cohort of TCGA-BLCA dataset, the risk model was constructed. By performing univariate Cox regression analysis for each variable, we found that a total of 29 variables were significantly associated with the prognosis of BLCA patients. To establish our risk model, Cox stepwise regression was used to further screen the variables. Finally, a model with 15 variables was selected. Risk score of each sample is shown in Table S6. As shown in Table S7, the high expression levels of C5AR2, CSF3R, FBXW10, FCAR, GHR, OLR1, PGLYRP3, RASGRP4, S100A12 and high value of Macrophages M1 were high risk factors for the prognosis of BLCA patients and predicted a poor prognosis. On the contrary, high expression levels of CD96, IL10, MEFV and high values of T cells CD8 and Eosinophils were associated with good prognosis. We divided the samples into high-risk group and low-risk group according to whether their risk score was greater than zero. Fig. 4A suggested that patients in high-risk group had significantly worse outcomes than those in low-risk group (*P* < 0.0001). Then, we used the ROC curve to test the prediction efficiency of the risk model. Fig. 4B showed the area under the curve (AUC) value of our model was 0.727, which indicated that our model has high reliability. Since bladder cancer accounts for about 90% of urothelial cancer, we used the Human Protein Atlas (HPA) database (https://www.proteinatlas.org/) to preliminarily verify the expressions of these genes in urothelial carcinoma tissues and their impact on the prognosis of patients. AS showed in Fig. 5, C5AR2, CSF3R, FCAR, GHR, PGLYRP3 and S100A12 were highly expressed and CD96, IL10 were lowly expressed in cancer tissues. Fig. 6 revealed that high expression of C5AR2, CSF3R, GHR, IL10, PGLYRP3, RASGRP4 were significantly associated with poor prognosis, whereas high expression of CD96 was significantly associated with good prognosis (*P* < 0.05). Except that the influences of the expression levels of FBXW10 and IL10 on prognosis were inconsistent with our conclusion, the other results above all confirmed our view.

### Inspection of the risk model through internal and external validation cohort

**Fig. 7:**
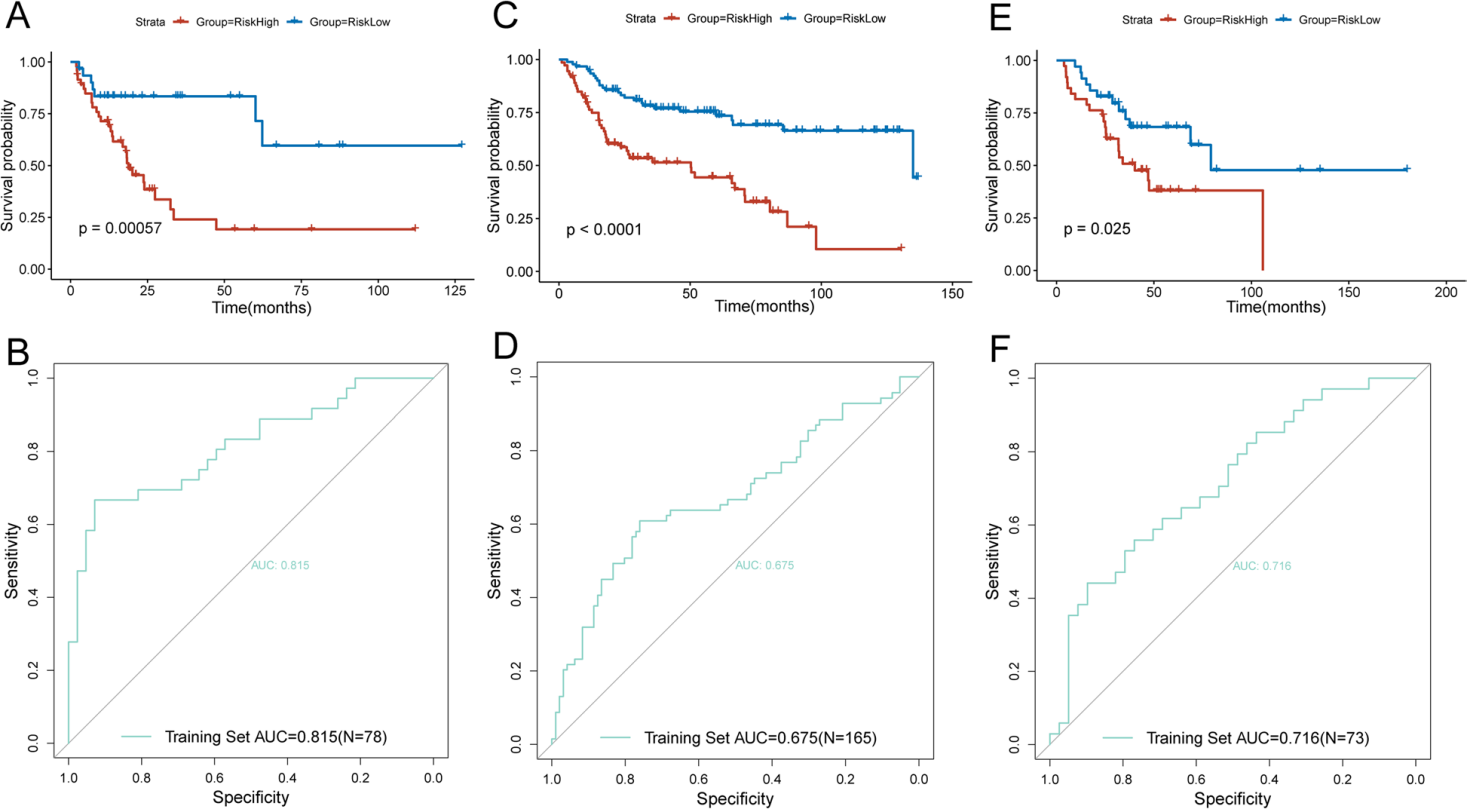
Internal and external validation of the risk model. **A** KM curves of the OS in the internal validation cohort. **B** ROC curves and AUC in the training cohort of the risk model. **C** KM curves of the OS in the GSE13507 validation cohort. **D** ROC curves and AUC in the GSE13507 validation cohort of the risk model. **E** KM curves of the OS in the GSE48276 validation cohort. **F** ROC curves and AUC in the GSE48276 validation cohort of the risk model

First, we used the internal validation cohort of the TCGA-BLCA dataset to check the usability of the risk model. As shown in Fig. 7A, among the samples in the internal validation cohort, there was a significantly difference in prognosis between the high-risk and low-risk groups (*P* < 0.001), and the low-risk group was better than the high-risk group. Then, we drew ROC curve of the risk model, AUC of the model was 0.815 (Fig. 7B).

In order to make our model more reliable, we used two external datasets GSE13507 and GSE48276 to further verify the model. Fig. 7C and Fig. 7E suggested that, the prognosis of low-risk group and high-risk group were also significantly different. Besides, AUC of the two cohorts were above 0.675 and 0.716, respectively (Fig. 7D and Fig. 7F).

### Construction of nomogram

**Fig. 8:**
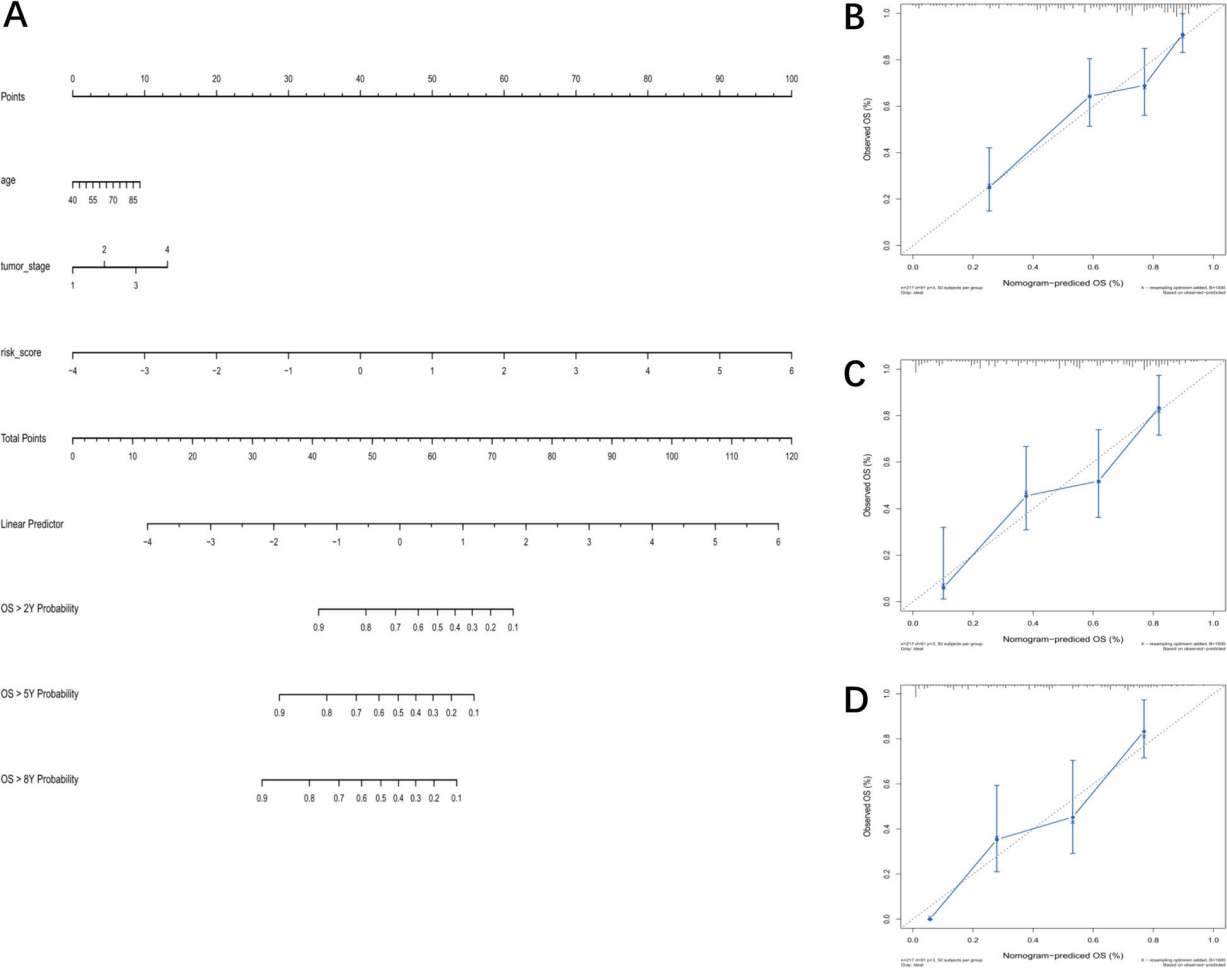
Construction and robustness verification of Nomogram. **A** Nomogram predicting the OS in BLCA patients containing the risk score. **B-D** Calibration curve of 2-year, 5-year and 8-year survival in the nomogram and ideal model

We screened for prognostic risk factors from clinical information collected from the training cohort samples. After univariate Cox regression analysis, Age, tumor stage, N stage and risk score were found to be risk factors for OS (*P* < 0.01). Then we further performed Cox stepwise regression analysis on the above four risk factors, and the results showed that age, tumor stage and risk score were independent risk factors for OS. The nomogram was shown in Fig. 8A. In order to confirm the accuracy of our nomogram, we reckoned its C-index value, which was 0.74. Moreover, Fig. 8B-Fig. 8D show the calibration curves of 2-year,5-year and 8-year survival between true situation and our nomogram. The results show that our model has relatively high accuracy in predicting the 2-year, 5-year, and 8-year survival rate of BLCA patients.

## Discussion

The number of BLCA patients worldwide is increasing, and the prognosis of bladder cancer patients varies according to race, gender. With the continuous development of molecular biotechnology in the field of oncology, we look forward to finding more accurate markers for predicting patient prognosis at the molecular level.

As we all know, immunity and autophagy play important roles in tumor formation and progression. For one thing, the immune system monitors and eliminates tumor cells, which is essential to prevent the occurrence and development of tumors. For another, with the development of tumors and other factors, the immune system’s ability to eliminate tumor cells decreases. Autophagy controls tumor cell proliferation and inhibits angiogenesis to achieve tumor suppressor effect. Meanwhile, autophagy may increase the stress ability of tumor cells to help them escape from the dead.

In the present study, patients with BLCA were divided into 6 molecular subtypes based on 2208 immune and autophagy-related genes. These 6 subtypes had significant differences in prognosis. For further analysis, we combined the 6 subtypes into two groups with significantly different prognosis based on the prognosis, and identified 65 DEGs in these two groups. The enrichment analysis of GO and KEGG revealed the DEGs were closely connected with the occurrence and development of tumors. Then, we established a prognostic risk model through univariate Cox and multivariate Cox stepwise regression analysis. According to internal and external validations, the risk model composed of C5AR2, CD96, CSF3R, FBXW10, FCAR, GHR, IL10, MEFV, OLR1, PGLYRP3, RASGRP4, S100A12, T cells CD8, Macrophages M1 and Eosinophils was stable and effective to predict BLCA patients’ prognosis. Finally, we further validated our model by observing the expression of above genes in tumor and normal tissues. Moreover, we established a nomogram that had a good prediction for the survival of patients with BLCA, which contained age, tumor stage and risk score. A high C-index value and a good calibration curve showed that the nomogram had a good predictive effect.

C5AR2 is a polyhedral modulator that can affect multiple systems and cell types, so it plays a dual role of immune activation and immune suppression [18, 19]. Several studies showed that C5AR2 can promote tumor formation and chemotherapy resistance by providing a living environment for cancer stem cells [20]. In general, C5AR2 is differentially expressed in most cancerous and noncancerous tissues, and high expression of C5AR2 is significantly connected with poor prognosis in many cancers. For example, Zhu et al. showed that overexpression of C5AR2 promoted the migration, invasion and proliferation of breast cancer cells [21]. In our research, high expression of C5AR2 was a risk factor for prognosis and was linked to poor prognosis in BLCA patients.

CD96 participates in a variety of immune responses, controls immune cell infiltration, and affects the malignant properties of various cancers. Thus, in various cancers, especially gliomas and melanomas, CD96 is a potential biomarker to determine patient immune infiltration and prognosis. High CD96 expression is associated with poorer overall and disease-specific survival in low-grade gliomas. But in cutaneous melanoma, the opposite correlation was found [22]. In the present study, overexpression of CD96 connected with good prognosis in BLCA patients.

Several studies showed that mutations of CSF3R are a risk factor of the development of myeloid and lymphoid malignancies [23]. Some studies have also suggested that CSF3R mutations may be effective diagnostic and prognostic markers for chronic neutrophilic leukemia and chronic myeloid leukemia [24, 25]. In our research, high expression of CSF3R contributed to poor outcomes of BLCA patients.

FBXW10 is an independent prognostic risk factor in hepatocellular carcinoma. High expression of FBXW10 is linked to poor survival in male hepatocellular carcinoma patients [26]. Wang et al. suggested that the mean methylation rate of FBXW10 in cancer tissues was significantly higher than in paired normal tissues in clear cell renal cell carcinoma [27]. In our study, high expression of FBXW10 would result in a high risk score with poor prognosis.

FCAR (CD89) mediates multiple immune system functions, including degranulation, endocytosis, phagocytosis, cytokine synthesis, and cytokine release [28]. As a regulator, FCAR plays a dual role of anti-inflammatory and pro-inflammatory in the inflammatory response [29]. Some researchers believed that FCAR was a promising therapeutic target for hematopoietic malignancies [30, 31]. Our study suggested that high expression level of FCAR led to poor prognosis in BLCA patients.

GHR may be implicated in many types of cancer. Studies related to gastric cancer have shown that GHR regulated the G1 cell cycle progression by mediating the PI3K/AKT signaling pathway, thereby regulating the growth and apoptosis of gastric cancer cells [32]. Strous et al. showed that dysregulation of GHR signaling was associated with cancer, and the GHR signaling pathway acted a vital role in growth, metabolism, immunity, cell cycle control, homeostatic processes, and chemoresistance through the JAK/STAT and SRC pathways [33]. Knockdown of GHR significantly stimulated apoptosis in gastric cancer cells, and resulted in arrest of the G1 cell cycle [32]. Our study suggested that GHR might also contribute to tumor development in BLCA.

IL10 is thought to have the ability to suppress antitumor T cell responses in cancer, but several researches have also suggested that IL10 took part in some inherent antitumor T cell responses. It indicates that IL10 may play a dual regulatory role in cancer [34]. In our study, IL10 played a certain antitumor effect in BLCA.

Studies related to chronic non-bacterial osteomyelitis indicated that the frequency of MEFV gene mutations increased in the disease, and the disease phenotype was more severe in patients with MEFV gene mutations [35]. In addition, studies have shown that mutations of MEFV gene which encode the pyrin protein could cause Familial Mediterranean fever [36]. Our study revealed that overexpression of MEFV was a favorable prognostic factor in BLCA patients.

LOX-1 encoded by the OLR1 gene is involved in the pathogenesis of atherosclerosis, and activation of LOX-1 is an important mechanism leading to plaque instability and progression to acute coronary syndrome [37]. Meanwhile, the upregulation of LOX-1 was associated with the occurrence, development and metastasis of various tumors [38]. In the present study, overexpression of OLR1 led to a higher risk score and a poorer prognosis, which was also consistent with the findings above.

At present, there are relatively few studies on PGLYRP3. According to research, PGLYRP3 acted a pivotal part in antibacterial immunity and inflammatory responses [39, 40]. In our research, PGLYRP3 A was highly expressed in BLCA and was associated with poor prognosis.

According to research, RASGRP4 was significantly overxpressed in diffuse large B cell lymphoma. Meanwhile, knockdown of RASGRP4 significantly inhibited tumor formation [41]. Studies on bladder urothelial carcinoma have found that overexpression of RASGRP4 was significantly related to shorter survival of bladder urothelial carcinoma [42]. Our study confirmed this.

S100A12 was proved to be a useful biomarker in inflammatory conditions. And some studies suggest that it might also take part in cardiovascular disease [43]. In cancer, S100A12 also played a regulatory role. For example, the expression of S100A12 was significantly upregulated in human papillary thyroid cancer, and knockdown of S100A12 significantly inhibited propagation, transfer, invasion, and cell cycle progression of cancer cells [44]. This study showed that high expression of S100A12 led to worse prognosis in BLCA patients.

Immunity and autophagy play important roles in tumors. Our study identified twelve genes associated with immunity and autophagy and three Cibersort immune infiltration scores that were significantly associated with bladder cancer prognosis. On this basis, we established a model to predict survival in patients with BLCA. There are some limitations to our study. First, the genes we defined were validated only by immunohistochemistry in the HPA database. Although the immunohistochemical data in the HPA database and the gene expression data in the TCGA are of relatively high quality, the data we used were from urothelial carcinoma and were not 100% representative of bladder cancer. Second, immunohistochemical information of FBXW10, MEFV, OLR1 and RASGRP4 were missing in HPA. The high expression of IL10 was detrimental to prognosis, which was inconsistent with our findings. Besides, the functions of these genes in bladder cancer need to be further explored. For BLCA patients with T1G3 stage, Bacillus Calmette-Guerin (BCG) treatment and response to BCG have important influence on the prognosis of patients. Unfortunately, our study did not have enough data at this point to make a credible statistical analysis, which was one of the limitations of this study. At the same time, genetic mutations may also have a significant impact on the prognosis of patients with bladder cancer. In many tumors, mutations in one or more genes have been shown to be significantly associated with prognosis. Unfortunately, we did not explore the genetic mutations in high-risk and low-risk patients.

## Supplementary Information

**Additional file 1: Table S1.** The difference of pTNM staging between high-risk group and low-risk group was significant.

**Additional file 2: Table S2.** The difference of immune infiltration index between high-risk group and low-risk group was significant.

**Additional file 3: Table S3.** 2208 immune-related and autophagy-related genes.

**Additional file 4: Table S4.** Differentially expressed genes.

**Additional file 5: Table S5.** GSEA KEGG enrichment.

**Additional file 6: Table S6.** Risk score of each sample from the training cohort.

**Additional file 7: Table S7.** Regression coefficient for each variable.

## Data Availability

The original contributions presented in the study are publicly available. These data can be found here: (https://www.ncbi.nlm.nih.gov/geo/), (https://portal.gdc.cancer.gov/), (https://reactome.org/).

## Abbreviations

TCGA: The Cancer Genome Atlas
BLCA: Bladder cancer
DEG: Differentially expressed gene
GEO: Gene Expression Omnibus
HPA: Human Protein Atlas
NMF: Non-negative matrix factorization
GO: Gene Ontology
KEGG: Kyoto Encyclopedia of Genes and Genomes
KM: Kaplan-Meier
MF: Molecular function
BP: Biological process
CC: Cellular component
BCG: Bacillus Calmette-Guerin
